# Characterization of Juquitiba Virus in *Oligoryzomys fornesi* from Brazilian Cerrado

**DOI:** 10.3390/v6041473

**Published:** 2014-03-26

**Authors:** Alexandro Guterres, Renata Carvalho de Oliveira, Jorlan Fernandes, Liana Strecht, Flavia Casado, Flavio Cesar Gomes de Oliveira, Paulo Sérgio D’Andrea, Cibele Rodrigues Bonvicino, Carlos Guerra Schrago, Elba Regina Sampaio de Lemos

**Affiliations:** 1 Laboratório de Hantaviroses e Rickettsioses, Instituto Oswaldo Cruz, Fundação Oswaldo Cruz, Rio de Janeiro, RJ CEP 21040-360, Brazil; E-Mails: reoliveira@ioc.fiocruz.br (R.C.O.); jorlan@ioc.fiocruz.br (J.F.); listrecht@ioc.fiocruz.br (L.S.); elemos@ioc.fiocruz.br (E.R.S.L.); 2 Departamento de Genética, Universidade Federal do Rio de Janeiro, Rio de Janeiro, RJ CEP 21941-570, Brazil; E-Mail: carlos.schrago@gmail.com; 3 Laboratório de Biologia e Parasitologia de Mamíferos Silvestres Reservatórios, Instituto Oswaldo Cruz, Fundação Oswaldo Cruz, Rio de Janeiro, RJ CEP 21040-360, Brazil; E-Mails: flaviacasado@gmail.com (F.C.); flaviocesar13@gmail.com (F.C.O.); dandrea@ioc.fiocruz.br (P.S.D.); cibelerb@inca.gov.br (C.B.); 4 Programa de Genética, Instituto Nacional de Câncer, Ministério da Saúde, Rio de Janeiro, RJ CEP 20230-092, Brazil

**Keywords:** Juquitiba virus, host switching, hantavirus cardiopulmonary syndrome, *Oligoryzomys fornesi*

## Abstract

The Juquitiba virus, an agent of Hantavirus Cardiopulmonary Syndrome, is one of the most widely distributed hantavirus found in South America. It has been detected in *Oligoryzomys nigripes, Akodon montensis, Oxymycterus judex, Akodon paranaensis* in Brazil and in *O. nigripes, Oryzomys* sp*.* and *Oligoryzomys fornesi* rodents in Argentine, Paraguay and Uruguay. Here, we report the genomic characterization of the complete S segment from the Juquitiba strain, isolated from the lung tissues of *O. fornesi*, the presumed rodent reservoir of Anajatuba virus in Brazilian Amazon, captured in the Cerrado Biome, Brazil.

## 1. Introduction

The hantavirus is among the most important zoonotic pathogens of humans, associated with rodents, insectivores and bats, and is the subject of studies worldwide. These RNA viruses of the family *Bunyaviridae* are the etiologic agents of hemorrhagic fever with renal syndrome (HFRS) in Europe and Asia, and hantavirus cardiopulmonary syndrome (HCPS), in the Americas [1,2]. Unlike other members of the Bunyaviridae, hantaviruses are not vector-borne but are instead transmitted between their vertebrate hosts through aggressive interactions or the inhalation of excreta [1].

The South American hantavirus is harbored by more than 27 species of rodents from the subfamily Sigmodontinae [3]. Brazil exhibits a high biodiversity, supporting approximately 450 of the 540 known species of *Sigmodontinae* rodents [4,5]. An increased number of eco-epidemiological studies have been conducted in different Brazilian regions and nine different hantavirus genotypes have been identified so far; some of them have been found to naturally infect more than one species of rodent. Cross-species transmission events at the genus and species levels has been suggested between South American rodents species, as reported for Juquitiba (JUQ) and Jabora (JAB), pathogenic and non pathogenic viruses, respectively [6,7,8,9]. We report here the JUQ virus in *Oligoryzomys fornesi,* the presumed reservoir for Anajatuba (ANAJ) virus in Brazilian Amazon, captured in Mato Grosso do Sul State, Cerrado Biome (Midwestern) [10]. Additionally, we conducted the genomic characterization of the complete S segment (1.9 kb) and fragments of the M segment (520 nt) from the JUQV strain, aiming towards a better characterization of the hantaviruses circulating in this rodent species.

## 2. Experimental

### Ethics Statement

Each animal was anesthetized and euthanized by intramuscular injection of sodium pentobarbital, all efforts were made to minimize suffering. Permits for field collection were granted by IBAMA (Brazilian Institute of Environment and Renewable Natural Resources, Rio de Janeiro, Brazil) permanent license under process number 13373-1.

**Figure 1:**
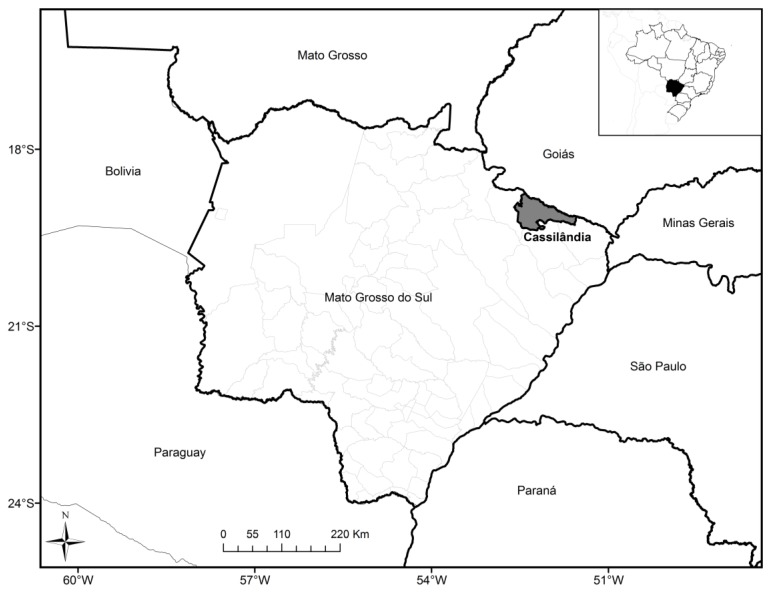
Map of the Mato Grosso do Sul State, Midwestern Brazil, showing the municipality of Cassilândia.

During an eco-epidemiological study performed in the Municipality of Cassilândia, Mato Grosso do Sul (Figure 1), in 2008, a total of 142 rodents were captured, with *Necromys lasiurus* being the most abundant species (56 specimens), followed by *Calomys explusus* (44 specimens), *Oligoryzomys fornesi* (10), *Cerradomys marinhus* (08), Calomys sp. (07), *Hylaeamys megacephalus* (05), *Nectomy rattus* (4), *Mus musculus* (03), *Cerradomys maracajuensis* (02), *Oligoryzomys* sp. (01), *Cerradomys sp.* (01), and *Oecomys* sp. (01). Two rodents of the species *O. fornesi* (Olfo_12070, Olfo_12071) were antiboby-reactive, with a seroprevalence of 1.41%. The genomic RNA was extracted from lung tissue samples of the two rodents according to the manufacturer´s instructions from the PureLink® Micro-to-Midi total RNA purification kit (Invitrogen, San Diego, CA, USA). The cDNA was then prepared with the SuperScript III First-Strand Synthesis System (Invitrogen) using specific primer (AG01-27F 5'-TAGTAGTAGACTCCTTGAKAAGCTACT-3'), and PCR was performed as described previously, using an extensive panel of oligonucleotides [11]. Both specimens were identified initially using external and cranial morphological analysis, and the identification was confirmed by kariologycal.

Multiple sequence alignment and comparison of nucleotide were performed using MUSCLE, in the SeaView v.4 software program [12]. Phylogenetic relationships were estimated using a Bayesian Markov Chain Monte Carlo (MCMC) method implemented in MrBayes v3.1.2 [13], using the GTR + G model of nucleotide substitution. MCMC settings consisted of two simultaneous independent runs with 4 chains each that were run for 10 million generations and sampled every 100th generation, yielding 100,000 trees. After eliminating 10% of the samples as burn-in, a consensus tree was built. Statistical support of the clades was measured by the approximate likelihood ratio test [14] and the Bayesian posterior probabilities. For analyses, sequences of Hantaan virus (NC005218 and EU363817) and Seoul virus (AY027040 and DQ133505) were used as outgroup species.

## 3. Results and Discussion

Originally, the *O. fornesi* specie was found naturally hosting the JUQV in Paraguay [15,16] and afterwards also in Northeast Brazil (3°16'S, 44°37'W), about 3,000 km of distance, hosting the Anajatuba virus genotype [10]. In Brazil, this of rodent species mainly occurs in open vegetation formations but can also be found in forest formations as the Cerrado and the Amazon region. These rodents have peridomestic affinities, being captured in and around human habitations. Although *O. fornesi* species has a wide geographic distribution, it may be common but not abundant, playing an important role in the maintenance of the hantavirus in South America [10,17]. 

**Figure 2:**
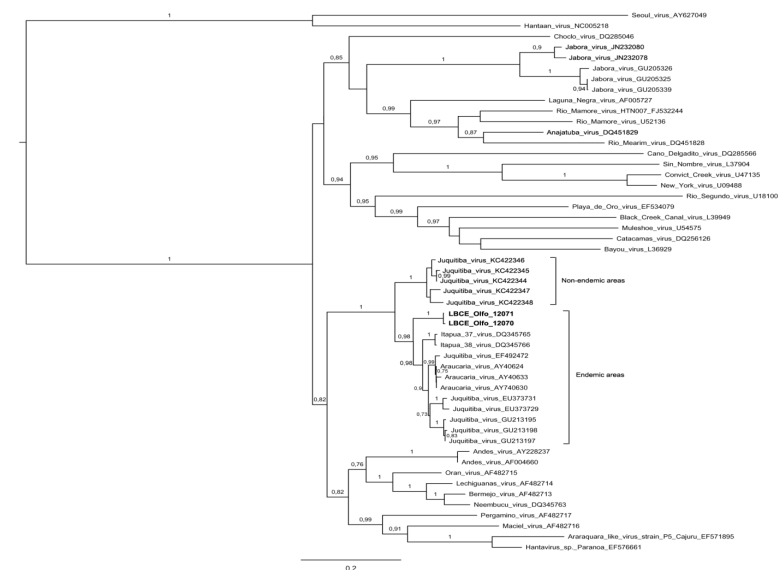
Phylogenetic relationships among hantaviruses based on a Bayesian analysis of genetic distances generated from comparisons of the nucleocapsid protein gene partial sequences (935 nt). The scale bar indicates a sequence divergence of 0.2. The numerical value at the node indicates the posterior probability (pp) replicates that supported the interior branch. The branch labels include GenBank^®^ accession number and viral species or strain (Access number: Genbank^®^ LBCE_Olfo_12070-KF913849, LBCE_Olfo_12071-KF913850).

Recently, two distinct genetic lineages in JUQV were proposed, one related with endemic areas and other associated non-endemic areas [11]. The new Mato Grosso do Sul State strains obtained from *O. fornesi* samples, together with the all the sequences of other JUQV strains, form a well-supported clade (branch support = 1), with nucleotide diversity ranging from 0.3%–12.7%. The amino acid sequences comparison revealed little inter-variability among groups of JUQV, with 3.8%. When compared with the ANAV sequence, the percentage of nucleotide diversity reached 24.7% and the amino acid sequences 13.5%, respectively. In the phylogenetic analysis based on partial S segment indicated that the hantavirus strain circulating in *O. fornesi* (Olfo 12070 and Olfo 12071) they are closely to sequences JUQV associated with endemic areas (Figure 2). With regard to host, the JUQV sequence in *O. fornesi* obtained in the Paraguay (Access number: Genbank^®^ GU213198) lies on the other end of the clade. These findings provide additional evidence that spillover infection of JUQV-related viruses is probably actively occurring among oryzomyine rodent species, as reported by Chu and collaborators [15,16]. 

**Figure 3:**
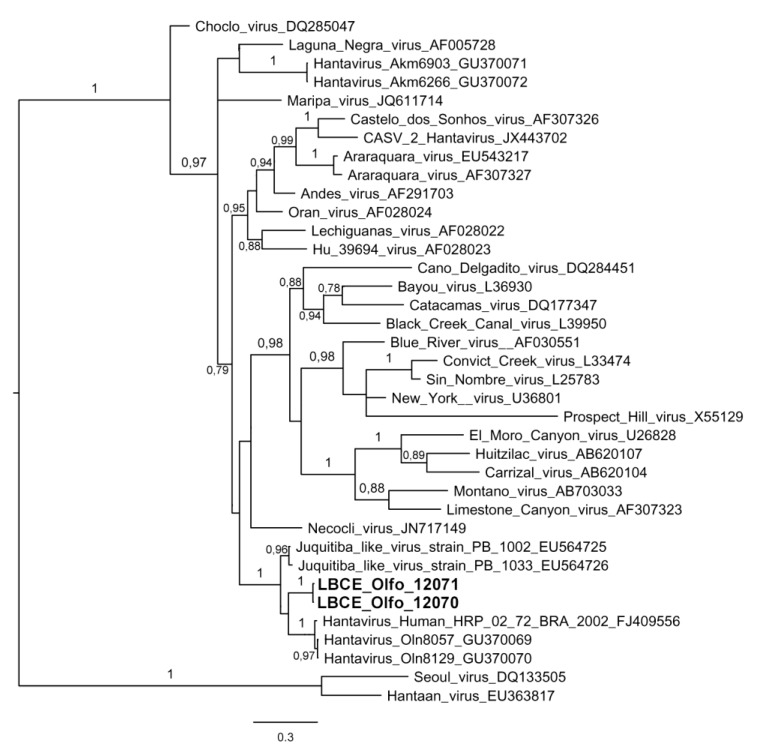
Phylogenetic relationships among hantaviruses based on a Bayesian analysis of genetic distances generated from comparisons of the glycoprotein gene (Gc) partial sequences (425 nt). The scale bar indicates a sequence divergence of 0.3. The numerical value at the node indicates the posterior probability (pp) replicates that supported the interior branch. The branch labels include GenBank^®^ accession number and viral species or strain (Access number: Genbank^®^ LBCE_Olfo_12070-KF913851, LBCE_Olfo_12071-KF913852).

A bayesian analysis based on partial M segment sequences (Figure 3) showed that the sequences obtained from two *O. fornesi* (Olfo 12070 and Olfo 12071) form a strongly supported clade (pp = 1), together with the sequences of other JUQV strains. The clade share a common ancient ancestor and is formed by sequences from human (Access number: Genbank^®^ FJ409556) and rodent samples of the specie *Olygoryzomys nigripes* (GU370069 and GU390070) from Brazil and sequences recovered from other rodents species, *Oxymycterus nasutus* (EU564725) and *O. nigripes* (EU564726) captured in Uruguay. The nucleotide diversity between JUQV ranged from 0.01% to 15.4%. The amino acid sequences comparison revealed little inter-variability among groups of JUQV, with 1.4%. We were not able to compare our sequences with the ANAV because there is a lack of available sequences in GenBank^®^. More investigations evaluating the M and L segments of JUQV genotype should be performed, to address relevant questions related to reassortment and recombination events. However, attention should be paid in the bioinformatic analysis, because there is a small number of JUQV sequences currently deposited in GenBank^®^, which prevents conclusive results for these events, as shown here and for Jabora genotype [18].

However, the close phylogenetic relationships among some hantavirus taxa across large geographic areas, and infected related hosts, supports the occurrence of virus-host codivergence [19,20], the scenario of hantaviruses transmission in South America could be more complex than previously thought. The doctrine of one specific rodent species as elemental reservoir for a specific hantavirus has been increasingly challenged. New hantaviruses have been discovered in shrews, moles and bats and the evidences points at host-switching events and co-circulation in multiple sympatric reservoir species, challenging the strict rodent-virus co-evolution theory [20,21]. Cross-species transmission is a significant process during spread, emergence, and evolution of RNA viruses [22]. Hantavirus spillover is more likely to occur with host populations inhabiting sympatric or syntopic regions, and cross-species transmission would presumably have greater chances of success if the host species are closely related [23].

The Laguna Negra (LAN) virus is a good example of cross-species transmission, with apparent success in the process during the spread of this virus. Initially, LANV was detected in the Chaco region of Paraguay, associated with a HCPS outbreak and a direct genetic link was established between the virus detected in the HCPS cases and in *Calomys laucha* rodents, implicating them as the primary rodent reservoir for LAN virus in Paraguay [24]. Posteriorly, in the hantavirus-endemic area of northwestern Argentina the LAN virus was recovered from human cases and from *Calomys callosus* samples. The high sequence identity between human and rodent samples implicated *C. callosus* as the primary rodent reservoir for LAN virus in Argentina [25]. Recently, phylogenetic analysis of partial sequences of the N gene showed LAN virus as the cause of HCPS, and the possible association of the organism with *Calomys callidus* rodents in western Brazil [26]. This showed that the LN virus was able to establish a successful relation with different *Calomys* species. 

The subfamily Sigmodontinae is one of the more diverse groups of mammals. The high diversity of these rodents, coupled with a recent distribution pattern that began in the northern Andes (Colombia and Venezuela), and later inhabited the more southern regions of South America [27,28,29]. The occurrence of virus adaptation and host switching appears to be more frequent in the Midwest and Southern regions of Brazil, extending to Paraguay, Uruguay and Argentina [8,16,30]. Additionally, considering that the hantavirus are RNA virus and are very recent according to Ramsden [31], this high diversity and recent radiation of the rodents in South America make these events (host switching and adaptation) more common than previously believed.

The JUQV genotype has being detected in many countries: Brazil, Argentina, Paraguay and Uruguay, in HCPS endemic and non-endemic areas, with a wide host range: *O. nigripes*, *O. fornesi*, *Akodon montensis, A. paranaensis, Oxymycterus judex* and *Oryzomys* sp. [7,15,18,32,33]. These observations, together with the data presented here, demonstrate that this hantavirus is very well established in South America, being found in a vast range of potential rodents reservoirs. It can be used as a very interesting model for understanding the relationship of hantaviruses and their Sigmodontinae rodent hosts. 

## 4. Conclusions

Infection of the same rodent species, as described here for *O. fornesi*, by distant hantaviruses, could be interpreted as incidental, and suggestive evidence that the JUQ virus can establish productive infections in this species requires further investigation. The capacity of these viruses to adapt to a new reservoir has been more frequent than what was previously thought, demonstrating how recent the introduction of this virus in the South America can be. More studies must be conducted in these areas in order to better understand the hantavirus adaptation process, and its implications on the dynamic and maintenance of the enzootic cycles. 
